# Organ Stiffness in the Work-Up of Myelofibrosis and Philadelphia-Negative Chronic Myeloproliferative Neoplasms

**DOI:** 10.3390/jcm9072149

**Published:** 2020-07-08

**Authors:** Edoardo Benedetti, Rita Tavarozzi, Riccardo Morganti, Benedetto Bruno, Emilia Bramanti, Claudia Baratè, Serena Balducci, Lorenzo Iovino, Federica Ricci, Vittorio Ricchiuto, Gabriele Buda, Sara Galimberti

**Affiliations:** 1 Department of Clinical and Experimental Medicine, UO Haematology, Azienda Ospedaliero-Universitaria Pisana, 56127 Pisa, Italy; ritatavarozzi@gmail.com (R.T.); claudia.barate@gmail.com (C.B.); s.balducci811@gmail.com (S.B.); liovino@fredhutch.org (L.I.); federica0189@gmail.com (F.R.); ga.buda@libero.it (G.B.); sara.galimberti@med.unipi.it (S.G.); 2 Section of Statistics, Azienda Ospedaliero-Universitaria Pisana, 56127 Pisa, Italy; r.morganti@ao-pisa.toscana.it; 3 Department of Molecular Biotechnology and Health Sciences, University of Turin, 10126 Torino, Italy; benedetto.bruno@unito.it; 4 Institute of Chemistry of Organo Metallic Compounds (ICCOM), CNR, Via G Moruzzi 1 56124 Pisa, Italy; bramanti@pi.iccom.cnr.it; 5 Dipartimento di Tecnologie Sanitarie ESTAR, Tecnologie Sanitarie, Azienda Ospedaliero-Universitaria Pisana, 56127 Pisa, Italy; ing.vricchiuto@libero.it

**Keywords:** myeloproliferative neoplasms, splenic stiffness, spleen, fibrosis, ultrasound

## Abstract

To define the role of spleen stiffness (SS) and liver stiffness (LS) in myelofibrosis and other Philadelphia (Ph)-negative myeloproliferative neoplasms (MPNs), we studied, by ultrasonography (US) and elastography (ES), 70 consecutive patients with myelofibrosis (MF) (no.43), essential thrombocythemia (ET) (no.10), and polycythemia vera (PV) (no.17). Overall, the median SS was not different between patients with MF and PV (*p* = 0.9); however, both MF and PV groups had significantly higher SS than the ET group (*p* = 0.011 and *p* = 0.035, respectively) and healthy controls (*p* < 0.0001 and *p* = 0.002, respectively). In patients with MF, SS values above 40 kPa were significantly associated with worse progression-free survival (PFS) (*p* = 0.012; HR = 3.2). SS also correlated with the extension of bone marrow fibrosis (BMF) (*p* < 0.0001). SS was higher in advanced fibrotic stages MF-2, MF-3 (W.H.O. criteria) than in pre-fibrotic/early fibrotic stages (MF-0, MF-1) (*p* < 0.0001) and PFS was significantly different in the two cohorts, with values of 63% and 85%, respectively (*p* = 0.038; HR = 2.61). LS significantly differed between the patient cohort with MF and healthy controls (*p* = 0.001), but not between the patient cohorts with ET and PV and healthy controls (*p* = 0.999 and *p* = 0.101, respectively). We can conclude that organ stiffness adds valuable information to the clinical work-up of MPNs and could be employed to define patients at a higher risk of progression.

## 1. Introduction

Philadelphia-negative chronic myeloproliferative neoplasms (Ph-neg MPNs) include clinical entities, polycythemia vera (PV), essential thrombocythemia (ET), and myelofibrosis (MF), with very different clinical manifestations and prognoses. Moreover, MF can present de novo as primary MF (PMF) or secondary to a prior MPN (either post-ET or post-PV) [1]. PMF prognosis is currently based on three scoring systems: the International Prognostic Scoring System (IPSS) [2], the Dynamic International Prognostic Scoring System (DIPSS) [3], and the DIPSS-plus [4]. Other novel prognostic systems include GIPSS [5] and MIPSS70 [6] and MIPSS70+ version 2.0 [7]. However, one limitation is the exclusion of the well-known prognostic role of common signs of progression, such as massive splenomegaly and marrow fibrosis [8,9]. 

Ultrasound sonography (US) has been widely used in clinical practice for more than 40 years in several clinical conditions. Nevertheless, it lacks quantitative information on tissue elastic properties [10]. More recently, elastography (ES) has allowed the in vivo assessment of soft tissue stiffness. It is based on the assumption that diseased tissues become harder than their healthy counterparts [11,12]. In our study, using conventional US and ES with the same sonographer, we concurrently assessed spleen and liver dimensions and their stiffness in patients with MPNs and in healthy volunteers. Our major aims were to investigate whether patient-specific characteristics, such as spleen stiffness (SS) and liver stiffness (LS), correlated with marrow fibrosis and could be predictive of clinical outcomes in MPN subtypes, with an emphasis on PMF.

## 2. Experimental Section: Patients and Methods

### 2.1. Patients

We included 87 MPN patients diagnosed and in follow-up (6-243 months from diagnosis) at our Hematology Unit of the University of Pisa, Italy. Seventy patients out of 87 gave their consent to the ES and US follow-up. From July 2018 to August 2019, 70 consecutive patients (dynamic cohort) with MPNs were enrolled in the US and ES study (Table 1). Follow-up ended in May 2020. Twenty healthy volunteers, from medical and nurse staff, were also enrolled as controls. They all tested negative for hepatitis B and C and HIV 1-2 blood serology, and had no medical history of spleen and/or liver abnormalities [13], portal vein dilation, and/or hematological disorder, as described in a recent report [14]. The study was approved by the Ethical Committee (n.12161, 3 March 2020). All patients and controls gave written consent upon enrollment. 

### 2.2. Methods

#### 2.2.1. Ultrasonographic Examination

**Figure 1:**
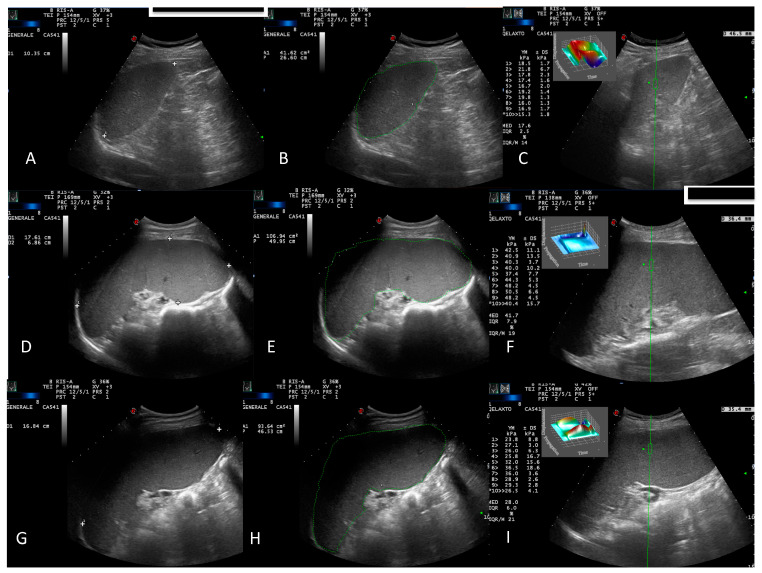
Spleen dimensions in a healthy control: splenic longitudinal diameter (SLD) (10.35 cm) (**A**), cross-sectional area (CSA) (41.62 cm^2^) (**B**), and splenic stiffness (SS) (17.6 kPA, IQR/M 14) (**C**). Spleen dimensions before and 1 year after treatment with Ruxolitinib in a patient with myelofibrosis: SLD (17.61 cm) (**D**), CSA (107 cm^2^) (**E**), and SS (41.7 kPA, IQR/M 19) (**F**); and SLD (16.85 cm) (**G**), CSA (93.64 cm^2^) (**H**), and SS (28 kPA, IQR/M 21) (**I**), respectively.

B-mode US, Doppler US, and point shear wave elastography (pSWE) were performed by the same physician at study entry and then every 3 months. US was performed with an Esaote Class-C-Advance ultrasonographer equipped with pSWE. A 1–5 MHz convex probe was used to assess abdominal organs. US and Doppler-US abdominal assessment included the following:

1. B-mode US evaluation of the liver, spleen, kidneys, gallbladder, pancreas, bladder, and retroperitoneal and splanchnic abdominal vessels (splenic and portal veins) [15]. US measurements of the liver and spleen were performed and expressed in cm, as previously described [16]. A patient’s spleen was evaluated in supine decubitus through the intercostal window. The US window, which included the splenic hilum, was considered optimal for biometric measurement of the organ. Measurements of the splenic longitudinal diameter (SLD) and cross-sectional area (CSA) were expressed in cm and cm^2^, respectively (Figure 1A,B,D,E,G,H). An SLD up to 11–12 cm in the cranio-caudal length and a CSA < 45 cm^2^ were considered normal [17], while moderate and marked splenomegaly were defined as a CSA in the range of 45–65 cm^2^ and >65 cm^2^, respectively [18];
2. Portal vein diameter measured at the crossing point with the hepatic artery and expressed in mm [19];
3. Portal vein flow velocity (PVV), expressed in cm/sec, with an intercostal window and sampled at the hepatic hilum. The maximum velocity (Vmax) and mean velocity (Vm) were taken in all patients and controls [19].

#### 2.2.2. Elastosonographic Examination

Following US assessment, after at least 3 h of fasting [20,21], splenic and liver pSWE were performed by the same sonographer. Liver pSWE was performed in a supine position with the right arm in maximal extension. The transducer was positioned in the intercostal space to visualize the right liver lobe. Artifacts and large vessels were avoided. The region of interest (ROI) was placed a minimum of 1–2 cm beneath the liver capsule, preferably between the VII and VIII hepatic segment [22,23,24,25]. A transient breath hold (3 to 5 sec) in a neutral position was required. Each procedure required less than 5 min [26]. Splenic pSWE was performed in the supine position with the left arm in the maximum possible abduction to increase the intercostal acoustic window. The best ROI was at located the lower pole at least 1 cm from the capsule. Large vessels were avoided [27]. Ten pSWE measurements were obtained for each patient and control. Assessment was defined as reliable (according to the manufacturer’s recommendations) when the interquartile (IQR)/median (M) ratio of the 10 measurements was ≤30%. IQR/M > 30% was defined as a technical failure [21].

#### 2.2.3. Statistical Analysis

The treatment response was evaluated using the European LeukemiaNet criteria for PV, ET [28], and MF [29]. Categorical data were described by the absolute frequency, and continuous data by the median and interquartile range (IQR). To compare qualitative variables with quantitative SS variables, the Kruskal–Wallis test followed by comparisons with Bonferroni’s inequality or Mann–Whitney tests were used. Spearman’s correlation analysis was performed to compare quantitative variables with SS. All factors significant in the univariate analysis were analysed in a multivariate model by multiple linear regression (MLR). Besides splenic and liver stiffness, expressed in both percentiles and as “high” or “low” compared to the median values, other variables included gender, age, blood count values, ferritin [30], LDH, and the mutational status at diagnosis. In MF, “events” were considered to be the reappearance of splenomegaly (at least 5 cm from the costal arch sign) or the doubling of SLD if between a 5 and 10 cm baseline, or a 50% increase if the baseline SLD > 10 cm, or transformation into acute leukemia with over 20% blasts in the marrow or >20% in peripheral blood with WBC > 10,000/μL, confirmed two weeks apart. In PV and ET, “events” were considered to be the partial or complete loss of a response or transformation into acute leukemia. Survival curves were calculated by the Kaplan–Meier method and differences between curves and the Hazard Ratio (HR) were measured with the log-rank test. Significance was set at 0.05. All analyses were performed with SPSS technology, version 25.

## 3. Results

### 3.1. Study Population

MPNs included 43/70 (61.4%) MF, of which 26/43 (60%) were PMF and 17/43 (40%) were SMF; 17/70 (24.3%) PV; and 10/70 (14.3%) ET. The body mass index (BMI) was equally distributed in patients and controls (median BMI 25 (range 19–30) and 24 (range 19–31), respectively, *p* = 0.960). In MF patients, BMF of grade 1, 2, and 3 was present in 11/43 (25.6%), 13/43 (30.2%), and 19/43 (44.4%) patients, respectively. For the purpose of this study, BMF was divided into a pre-fibrotic/early fibrotic stage (MF-0, MF-1) and an advanced fibrotic stage (MF-2, MF-3) by the W.H.O. classification [1,31]. Forty-three patients of 70 (61.4%) reported MPN-related symptoms by applying the MPN10 score [32]. The karyotype was normal in 60/70 (86%) of patients and complex in 6/70 (8%), while in 4/70 (6%), other chromosomal abnormalities, such as del(20q), t(3; 3), +8, and +9, were observed. The JAK2(V617F) mutation was found in 86% of patients; CALR and MPL mutations were observed in 2% and 9%, respectively.

### 3.2. Splenic and Liver Assessment

The complete B Mode ultrasound and elastography parameters of splenic and liver assessments are reported in Table 2. We reported only one (5%) technical failure by pSWE in a healthy control similar to what was previously described [14].

**Figure 2:**
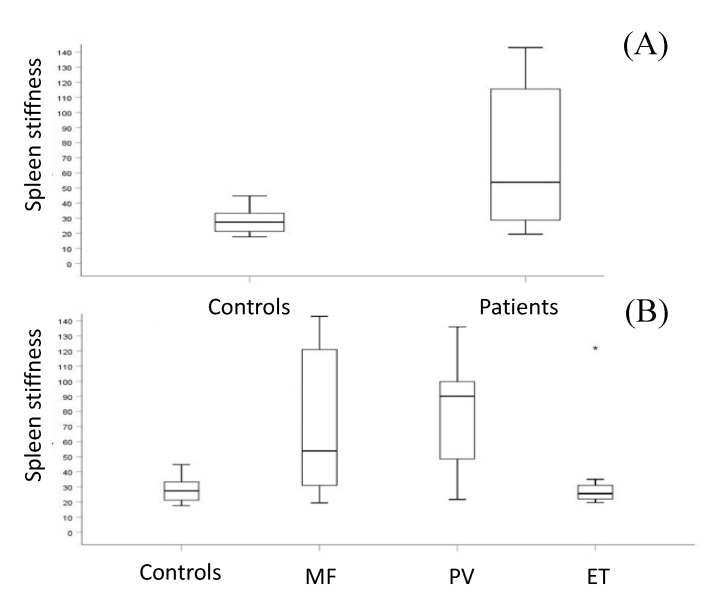
Comparisons of spleen stiffness (also see text): (**A**) Healthy controls vs. patients with myeloproliferative neoplasms (*p* < 0.0001); (**B**) healthy controls vs. myelofibrosis (MF) (*p* < 0.0001), healthy controls vs. polycythemia vera (PV) (*p* = 0.002), essential thrombocytopenia (ET) vs. MF (*p* = 0.014), and ET vs. PV (*p* = 0.027).

Univariate and multivariate analyses are presented in Table 3. In patients with MPNs, the median splenic LD, median CSA, and median SS were significantly higher than in healthy controls (*p* < 0.001, *p* < 0.001, and *p* < 0.001, respectively) (Figure 1A–F, Figure 2A). With regard to the diagnostic category, the median SS was not significantly different between patients with MF and PV (*p* = 0.9) whose SS was higher than healthy controls (*p* = 0.002). However, both MF and PV groups had significantly higher SS than the ET group (*p* = 0.011 and *p* = 0.035, respectively) whose SS did not differ significantly from healthy controls (*p* = 0.9) (Figure 2B). 

**Figure 3:**
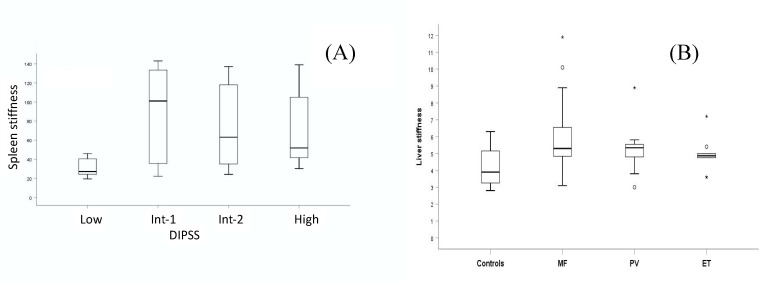
(**A**) Correlations of spleen stiffness with the Dynamic International Prognostic Scoring System (DIPSS). A trend between the low-risk and the intermediate (Int-1) group was reported (*p* = 0.059), while no difference was seen between the Int-1 and Int-2 (*p* = 0.541) groups, and the Int-1 and Int-2 with the high-risk group (*p* = 0.611 and *p* = 0.916). (**B**) Liver stiffness significantly differed between the patient cohort with MF and healthy controls (*p* = 0.001), but not between the patient cohorts with ET and PV and healthy controls (p = 0.999 and p = 0.101, respectively). Multiple comparisons did not show differences in liver stiffness between the different MPN categories (ET vs. MF, *p* = 0.440; ET vs. PV, *p* = 0.999; and MF vs. PV, *p* = 0.999).

In the patient cohort with MF, SS significantly correlated with the extension of BMF. The MF-2 + MF-3 group had higher SS than the MF-0 + MF-1 group (*p* < 0.0001). SS did not differ between primary and secondary MF (*p* = 0.329). No significant correlation was observed between SS and DIPSS subgroups. A trend between the low-risk and the intermediate (Int-1) group was reported (*p* = 0.059), while no difference was seen between the Int-1 and Int-2 (*p* = 0.541) groups, and the Int-1 and Int-2 with the high-risk group (*p* = 0.611 and *p* = 0.916) (Figure 3A). 

Multiple comparisons did not show differences in liver stiffness between the different MPN categories (ET vs. MF, *p* = 0.440; ET vs. PV, *p* = 0.999; and MF vs. PV, *p* = 0.999). Liver stiffness significantly differed between the patient cohort with MF and healthy controls (*p* = 0.001), but not between the patient cohorts with ET and PV and healthy controls (*p* = 0.999 and *p* = 0.101, respectively) (Figure 3B).

As demonstrated by the multivariate analysis, SS strongly correlated with BMF (*p* < 0.0001), while there was a trend for the association with liver LD (*p* = 0.067) and CSA (*p* = 0.095). Finally, no correlation was found between SS and the other studied variables.

### 3.3. Spleen Stiffness and Disease Progression

**Figure 4:**
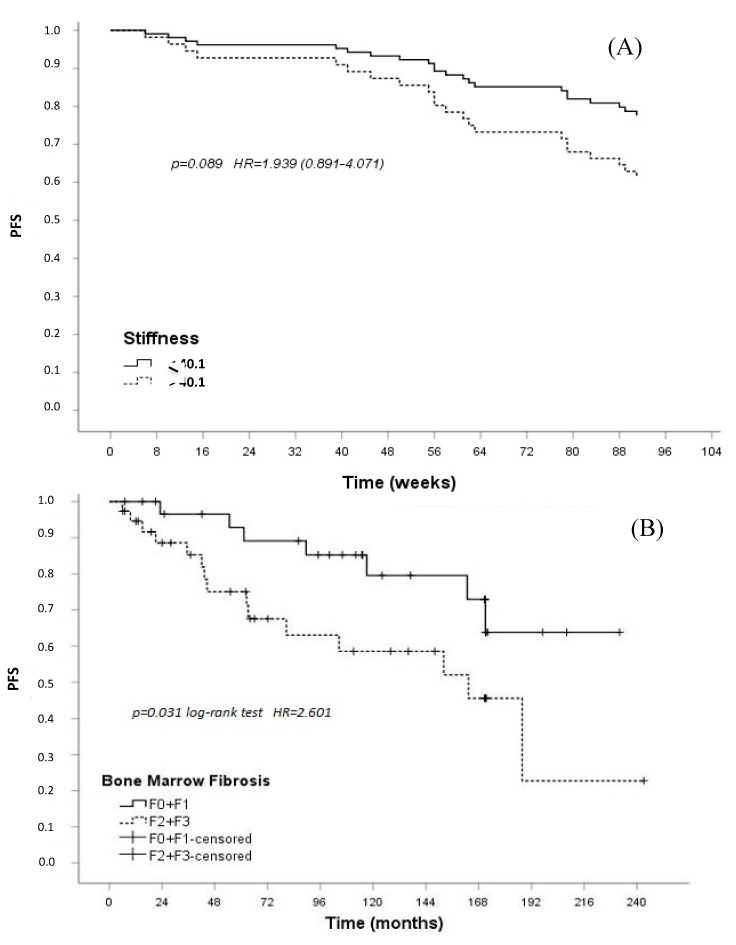
Spleen stiffness (SS) and bone marrow fibrosis (BMF) and progression-free survival (PFS) (Cox regression model) in patients with myelofibrosis. (**A**) PFS in patients with SS lower than the median value (40 kPa) (dotted line) vs. those with higher values (solid line) (*p* = 0.089; Hazard Ratio (HR) = 1.939 (range 0.891-4.071)). (**B**) PFS in patients with BMF grade MF-0, MF-1 (solid line) vs. those with BMF MF-2, MF-3 (dotted line). F0, F1, F2, and F3 in the legend represent MF-0, MF-1, MF-2, and MF-3, respectively.

To analyze the correlation between SS and survival (PFS from Jan 2018 to May 2020), univariate Cox analysis was performed, HR was calculated (1.939; CI 95%: 0.891–4.071), and a statistical trend was indicated (*p* = 0.089) (Figure 4A). Overall, at a median follow-up of 97 (range 6–243) months from diagnosis for the entire dynamic cohort, the median PFS of the whole population with MPNs was 188 months. At 8 years, 89% of the patients were progression-free, with a significant advantage for those with PV or ET compared to the cohort with MF (8-years PFS 100% vs. 80%; *p* = 0.044). It should be noted that all of the 21 progression events that occurred during the study period were observed in patients with primary MF (N = 17 increased splenomegaly, N = 3 peripheral blood blasts, and N = 1 bone marrow leukemic transformation, defined according to the IWG-MRT and ELN consensus report [29]). In this patient cohort, we tested with Kaplan–Meier quartiles, and we found that 40 kPa is the 50th (median) percentile with the best cut off.

In patients with BMF grade MF-0+MF-1 vs. MF-2+MF-3, PFS was 85% and 63%, respectively (*p* = 0.031; HR = 2.601) (Figure 4B).

### 3.4. Spleen Stiffness and Ruxolilitinib

Five patients, including four with MF and one with PV, on ruxolitinib, were assessed with B mode US and pSWE before the start of therapy and every 3 months thereafter. At a median follow-up of 9 months (range 7–13), in three patients with MF and in one with PV, the CSA shrank from 163 to 147 cm^2^ (10%), 107 to 94 cm^2^ (12%), 106 to 93.6 cm^2^ (11.7%), and 101 to 85 cm^2^ (15%), respectively. In the first three of these patients with MF, SS was also reduced from 143 to 81.9 kPa, 41.7 to 28.0 kPa, and 101 to 51.8 kPa, respectively (Figure 1E,F,H,I). In the other patients with MF and PV, both SS and CSA were still stable at the 1-year follow-up.

## 4. Discussion

MF, PV, and ET are classified under the W.H.O. category of myeloproliferative disorders. In the past decade, three prognostication systems (I.P.S.S., D.I.P.S.S., and D.I.P.S.S.-Plus) have been introduced for the risk stratification of patients with PMF. However, they fail to incorporate the prognostic role of disease manifestations, such as neutropenia, a cytokine profile, massive splenomegaly, or marrow fibrosis, whereas common signs of progression are indeed an increase in the severity of symptoms and worsening of splenomegaly [33].

Given the importance of organ involvement in PMF, and in MPNs in general, we investigated the role or organ dimensions and organ stiffness of the spleen and liver with two imaging techniques. The assessment of the liver and spleen size by B mode US is well-established in the work-up of hematological malignancies [16,34], while ES techniques have only recently been implemented in the clinical setting. Quantitative ES methods include transient ES (FibroScan^®^, Echosens, Paris, France), pSWE, and two-dimensional (2D)-SWE [14]. Shear wave elastography determines the mechanical properties of a tissue by monitoring the speed of shear waves generated by the ultrasound-induced acoustic radiation force. Ultra-sonographers can currently be equipped with software specific for ES. This integrated technology allows a multi-parametric assessment of both liver and splenic stiffness by the same sonographer employed for conventional B-mode and Doppler examinations [21]. ES has been used in the assessment of liver fibrosis, reducing the need for biopsies [24,35] and the work- up of liver cirrhosis complications, including portal hypertension [36,37]. However, only two studies have so far employed ES in patients with Philadelphia (Ph)-negative myeloproliferative neoplasms (Ph-neg MPNs) [1,2,31,38]. To the best of our knowledge, for the first time, we concurrently investigated the role of organ dimensions and organ stiffness in patients with Ph-neg MPNs. It should be noted that both Bimodal US and ES were performed by the same sonographer [14]. Clinical findings were also correlated with a “homogenous” cohort of healthy individuals, as defined by Giuffrè et al. [14]. As a whole group, patients with Ph-neg MPNs had a significantly higher SS compared to controls (Figure 2A,B). However, by disease subgroup, SS was significantly higher in patients with PV and MF, but not in those with ET. Accurso et al. [39] reported that palpable splenomegaly at diagnosis was found in 5–20% of ET patients and 31% of PV patients. Barraco et al. [40] found that 48% of PV patients had ≥grade 1 bone marrow reticulin fibrosis, and Iurlo et al. [31] previously showed a correlation between bone marrow fibrosis and SS in MF. All of these observations (frequency of splenomegaly and bone marrow fibrosis in MF and PV with respect to ET) could at least in part explain the difference in SS between MF and PV vs. ET found in our study.

There is increasing evidence that BMF has prognostic significance in PMF [8,9,41]. In our study, through multivariate analysis, SS significantly correlated with BMF. Therefore, we confirmed that SS, evaluated by pSWE, may serve as a surrogate marker of BMF. We can also speculate that the assessment of SS may drastically reduce the need for multiple invasive bone marrow biopsies [31]. Moreover, in our patients, we did not find a significant correlation between SS and scoring systems. A trend was observed between SS and the low-risk group; no differences were observed between SS and Int-1, Int-2, and the high-risk group (Figure 3A).

Given the correlation between SS and BMF, we also investigated the influence of SS on clinical outcomes from study entry. An SS higher than the median value of 40 kPa was associated with a higher risk of disease progression with an HR = 1.939 (Figure 4A). Therefore, SS does not strongly affect the PFS, but a statistical trend is present. This finding, if confirmed in larger studies with longer follow-up periods, may help clinicians to personalize patient follow-up and select those at a higher risk of progression who may benefit from earlier JAK inhibitor treatment [31]. Moreover, as previously reported by Abdel-Wahab et al. [42], no statistically significant difference in PFS was noticed between primary and secondary MF. 

BMF is usually progressive in MF. It may respond to interferon-alpha treatment in selected patients or even resolve with an allograft [43,44], while JAK inhibition may slow its progression [45]. A recent study showed an improvement in BMF of 35% with ruxolitinib compared with 3% using the best available therapy at 5 years of follow-up [46]. Iurlo et al. reported a concomitant reduction of SS and LD in three patients with MF treated with ruxolitinib [31]. In our study, we could assess five patients. Four patients treated with ruxolitinib showed a reduction of their splenomegaly (decrease in CSA of 10–15%), and three exhibited a decrease in SS, while in the remaining patients, both splenomegaly and SS stabilized. Though four out of five were on a reduced dose of ruxolitinib, ranging from 17% to 50% of the recommended dosage, due to compliance, none experienced further worsening of the splenomegaly at a median follow-up of 9 months from the start of treatment. Given our finding that SS correlates with BMF, it may be speculated that a decrease in SS while on ruxolitinib may also reflect a concurrent reduction in BMF. Though prospective control studies are warranted to confirm this hypothesis, the combination of SS by ES and spleen dimensions by B mode US may become dynamic parameters for evaluating treatment responses without the need for invasive bone marrow biopsies.

When organ stiffness parameters were correlated with organ dimensions, SS appeared to correlate more with CSA [18] than SLD [17]. This suggests that, in this setting, the CSA may better define organ biometric variations than the SLD. Future ultrasound studies that monitor SLD and CSA may prospectively evaluate the dynamic variations of spleen dimensions during the disease follow-up period and potentially define characteristic B mode US patterns that may correlate with early progression [18].

As in chronic liver diseases [24,37], we also assessed LS in MPNs and no significant differences were observed between subgroups. However, differences were noticed when the single subgroups were compared to healthy controls. Interestingly, LS in patients with MF was significantly higher, whereas in patients with PV and ET, it did not differ from healthy controls. If confirmed in a larger cohort of patients, it may be speculated that in a patient with MPNs with splenomegaly, LS may be indicative of MF rather than PV. Finally, there was a significant correlation between LS and SS when conducting univariate analysis (Table 2, *p* < 0.001), not confirmed by multivariate analysis (*p* = 0.478). Moreover, LS did not correlate with BMF.

It is worth pointing out that both a report by Iurlo et al. and ours showed rather similar findings with two ES techniques, consisting of fibroscan [31] and pSWE [27], respectively. However, we used a single sonographer. In general, elastography can be applied with a number of techniques and devices, manufactured by different companies, and have different reference values [27]. Our results may be used as a comparison/reference for future studies using the same sonographer or other ES techniques.

In conclusion, our study shows that SS, evaluated by pSWE, appears to be a reliable surrogate marker of BMF in MPNs. SS also displayed prognostic significance and correlated with PFS in patients with MF. Finally, SS may become a dynamic parameter to select patients at a higher risk of progression, who may potentially benefit from early intervention with ruxolitinib or alternative treatments such as an allograft.

## Figures and Tables

**Table 1:** Clinical and baseline patient characteristics.

**Age**	**68 (IQR ^1^: 53.5–76.0)**
**Sex**	
Male	35
Female	35
**Diagnosis**	
Primary MF	26 (41.3%)
Secondary MF	17 (20.3%)
PV	17 (24.3%)
ET	10 (14.3%)
**BMI (Body Mass Index)**	**24 (IQR 23–26)**
**Mutational status**	
JAK2 (V617F)	49 (70%)
CALR	11 (16%)
None	10 (14%)
**Kariotype**	
Normal	60 (87.1%)
Complex	4 (5.7%)
Other	6 (8.6%)
**Bone Marrow blasts (biopsy)**	**5% (3–5%)**
**BM fibrosis**	
0	3 (4.3%)
1	29 (41.4%)
2	19 (27.1%)
3	19 (27.1%)
**Symptoms**	
none	43 (61.4%)
**Blood test**	
WBC	
MF	8.120 × 10^9^/L (range 1.170–34.000)
ET	7.000 × 10^9^/L (range 5.000–15.700)
PV	9.000 × 10^9^/L (range 3.310–140.000)
**Hemoglobin**	
MF	12 gr/dL (range 8–18)
ET	12.9 gr/dL (range 10–15)
PV	18 gr/dL (range 9–21)
**Hematocrit**	
MF	36% (range 22–49)
ET	40% (range 36–47)
PV	53% (range 29–61)
**Platelet**	
MF	400.000 × 10^9^/L (range 55.000–900.000)
ET	700.000 × 10^9^/L (range 150–900.000)
PV	450.000 × 10^9^/L (range 90.000–913.000)
**LDH**	
MF	462 U/L (range 200–2630)
ET	243 U/L (100–550)
PV	263 U/L (100–450)
**Ferritin**	
MF	122 ng/mL (21–200)
ET	75.5 ng/mL (21–129)
PV	100 ng/mL (9–122)
**Patients treated with Ruxolitinib**	
Policitemia Vera	4 (18%)
Myelofibrosis	10 (82%)

^1^ IQR = Interquartile range.

**Table 2:** Splenic and liver assessment conducted by B Mode ultrasound and point shear wave elastography (pSWE).

Parameter	M Spleen LD	M Spleen CSA	M Splenic Stiffness	M Diameter of Splenic Vein	M Liver Stiffness	M Diameter of Portal Vein	Portal Vein Flow	Parameter
	(in cm)	(in cm^2^)	(in kPa)	(in mm)	(in kPa)	(in mm)	M Vmax/M Vm	
MPN	16.7	91.5	53.8	8	5 (r 4.5–6.9)	11	41.5/31.9	MPN
(70 pts)	(r 13.8–19.7)	(r 67.4–121)	(r 19.4–143)	(r 7–19)		(r 4–11)	(r 34–42)/(r 24–34)	(70 pts)
Healthy Controls	11.1	34	27.5	6.9	3.9	10.1	31.6/32	Healthy Controls
−20	(r 7.9–12.5)	(r 21–55)					(r 22.5–42.3)/(r 21.5–39.9)	−20
MF			53.96		5.3			MF
(43 pts)			(r 31–121)		(r 3.1–11.9)			(43 pts)
MF-0 MF-1			30.3					MF-0 MF-1
(11 pts)			(r 19.4–132)					(11 pts)
MF-2 MF-3			99.7					MF-2 MF-3
(32 pts)			(r 24.2–164.3)					(32 pts)
PMF			50.7					PMF
(26 pts)			(r 19.4–143)					(26 pts)
SMF			107					SMF
(17 pts)			(r 24–141)					(17 pts)
E.T.			25.6		4.95			E.T.
(10 pts)			(r 22–31)		(r 3.6–7.2)			(10 pts)
P.V.			90.1		5.3			P.V.
(17 pts)			(r 48.5–99.7)		(r 3–8.9)			(17 pts)

Abbreviations: MPN: myeloproliferative neoplasia; MF: myelofibrosis; ET: essential thrombocythemia; PV: polycythemia vera; PMF: primary MF; SMF: secondary MF; M: median; pts: patients; r: range; CSA: cross-sectional area; LD longitudinal diameter.

**Table 3:** Univariate and multivariate analysis of spleen stiffness vs. quantitative and qualitative variables. RC = regression coefficient.

	Univariate Analysis		Multivariate Analysis	
Variables	Rho or Median (IQR)	*p*-Value	RC	*p*-Value
**Age**		0.163		
Range: 68 years (IQR 53.5–76)	0.171			
**PV**		0.388		
(1) yes	90.1 (IQR 48.5–99.7)			
(0) no	46.5 (IQR 27.7–119.0)			
**ET**		0.076		0.121
(1) yes	25.6 (IQR 22.0–31.0)		−21.6	
(0) no	76.6 (IQR 35–121)			
**MF**		0.141		
(1) yes	53.9 (IQR 31.0–121.0)			
(0) no	45.5 (IQR 24.1–97.9)			
**BMI**		0.126		
Range: 24 (IQR 23–26)	0.187			
**Mutational status**		0.411		
(0) none	30.7 (IQR 26.3–71.6)			
(1) *JAK2* (V617F)	80 (IQR 30.6–115.5)			
(2) *CALR* or *MPL*	41.7 (IQR 31.7–124.5)			
**Karyotype**		0.338		
(0) missing value	92.1 (IQR 73.4–96.1)			
(1) favourable	47 (IQR 26.7–112)			
(2) unfavorable	108.3 (IQR 71.6–137)			
**Bone marrow blasts**		0.27		
Range: 5% (IQR 3–5%)	0.136			
**Bone marrow fibrosis**		**<0.0001**		**<0.0001**
Score: 0–4	0.584		22.8	
**Symptoms**		0.129		
(0) no	41.7 (IQR 28.3–119)			
(1) yes	73.2 (IQR 31–121)			
**White cell count**		0.177		
Range: 8505/mcL (IQR 5000–12,200)	0.166			
**Hgb**		0.725		
Range: 12.4 g/dL (IQR 10.6–15)	−0.043			
**HcT**		0.966		
Range: 38% (IQR 32.5–47)	−0.005			
**PLT**		0.102		
Range: 450,000/mcL (IQR 180,000–623,000)	−0.197			
**LDH**		0.187		
Range: 336 U/L (IQR 232–541)	0.191			
**Splenic LD**		0.211		
Range: 16.7 cm (IQR 13.8–19.7)	1.154			
**Splenic CSA**		0.080		0.095
Range: 91.5 cm^2^ (IQR 67.4–121)	0.211		−0.21	
**Liver LD**		0.001		0.067
Range: 16 cm (IQR: 15–18)	0.384		4.29	
**Splenic vein**		0.368		
Range: 8 mm (IQR 7–9)	0.111			
**Portal vein**		0.308		
Range: 11 mm (IQR 9–11.7)	−0.125			
**Mean velocity** Range: 31.9 cm/s (IQR 24.5–34)	0.156	0.17		
**Maximum velocity**		0.454		
Range: 41.5 cm/s (IQR 34–42)	0.092			
**Liver S**	0.455	<0.001	−22.0	0.478
